# DNA double-strand break repair is impaired in presenescent Syrian hamster fibroblasts

**DOI:** 10.1186/s12867-015-0046-4

**Published:** 2015-10-12

**Authors:** Ljudmila Solovjeva, Denis Firsanov, Anastasia Vasilishina, Vadim Chagin, Nadezhda Pleskach, Andrey Kropotov, Maria Svetlova

**Affiliations:** Institute of Cytology, Russian Academy of Sciences, 4 Tikhoretski ave., Saint Petersburg, 194064 Russia; Saint-Petersburg’s State Pediatric Medical University, Ministry of Health of Russian Federation, 2 Litovskaya st., Saint Petersburg, 194100 Russia

**Keywords:** Premature aging, Syrian hamster, Bleomycin, Double-strand break repair

## Abstract

**Background:**

Studies of DNA damage response are critical for the comprehensive understanding of age-related changes in cells, tissues and organisms. Syrian hamster cells halt proliferation and become presenescent after several passages in standard conditions of cultivation due to what is known as «culture stress». Using proliferating young and non-dividing presenescent cells in primary cultures of Syrian hamster fibroblasts, we defined their response to the action of radiomimetic drug bleomycin (BL) that induces DNA double-strand breaks (DSBs).

**Results:**

The effect of the drug was estimated by immunoblotting and immunofluorescence microscopy using the antibody to phosphorylated histone H2AX (gH2AX), which is generally accepted as a DSB marker. At all stages of the cell cycle, both presenescent and young cells demonstrated variability of the number of gH2AX foci per nucleus. gH2AX focus induction was found to be independent from BL-hydrolase expression. Some differences in DSB repair process between BL-treated young and presenescent Syrian hamster cells were observed: (1) the kinetics of gH2AX focus loss in G0 fibroblasts of young culture was faster than in cells that prematurely stopped dividing; (2) presenescent cells were characterized by a slower recruitment of DSB repair proteins 53BP1, phospho-DNA-PK and phospho-ATM to gH2AX focal sites, while the rate of phosphorylated ATM/ATR substrate accumulation was the same as that in young cells.

**Conclusions:**

Our results demonstrate an impairment of DSB repair in prematurely aged Syrian hamster fibroblasts in comparison with young fibroblasts, suggesting age-related differences in response to BL therapy.

**Electronic supplementary material:**

The online version of this article (doi:10.1186/s12867-015-0046-4) contains supplementary material, which is available to authorized users.

## Background

Replicative senescence was first described more than 50 years ago [1]. Telomeres of living cells progressively shorten during each cycle of replication due to the failure of DNA polymerase to extend lagging DNA strands in the absence of telomerase. It is believed that replicative senescence contributes to aging. Proteins p53, Rb and p16Ink4a (cyclin-dependent kinase inhibitor acting upstream of Rb) [2] are involved in this process. Various physiological stresses that are not accompanied by telomere shortening, e.g. the action of ionizing radiation (IR) and other DNA-damaging agents, oxidative stress, oncogenic stress (oncogene overexpression) also lead cells into a senescent state. These stresses induce cell cycle arrest which is followed by mTOR kinase activation necessary for progression of cellular senescence [3, 4]. General features of senescent cells include changes in morphology, chromatin organization and gene expression. It has been shown that senescent cells enhance the secretion of many inflammatory modulators and acquire p38 MAPK-dependent senescence-associated secretory phenotype (SASP) [5]. Rodent fibroblasts stop dividing after a very limited number of passages in culture due to oxidative stress induced by conventional culture conditions, although their telomeres remain almost unshortened [6]. This phenomenon is not observed in cultured human fibroblasts, which undergo senescence after an average of 50 population doublings. Proliferation arrest in rodent cells is due to standard culture conditions (20 % oxygen) and is termed «culture stress» [7]. Alternatively, mouse embryonic fibroblasts grown in culture conditions with 3 % O_2_ do not show the signs of premature aging [8].

The gradual accumulation of oxidative DNA damage occurs in all living organisms during their lifespan. It is induced by reactive oxygen species (ROS) mainly generated during normal activity of mitochondria. ROS-induced oxidative damages include apurinic/apyrimidinic DNA sites, oxidized purines and pyrimidines, single-strand DNA breaks (SSBs) and double-strand DNA breaks (DSBs) [9]. Numerous reports demonstrate an association of ROS-induced DNA damage and cellular senescence [10]. DNA damage is directly associated with the appearance of human diseases such as cancer and neurodegenerative disorders [11]. DNA repair capacity appears pivotal to the maintenance of genome integrity that makes the research of age-related aspects of DNA damage response (DDR) valuable.

We used Syrian hamster fibroblasts that prematurely stopped dividing in culture as a model for the study of the effect of chemotherapeutic drug bleomycin (BL) in non-replicating presenescent cells. BL is isolated from *Streptomyces verticillus* and belongs to a family of DNA-cleaving glycopeptides. BL is considered to be a radiomimetic agent because it produces lesions similar to those induced by IR. BL is used in combination therapy of lymphomas, testicular cancers and carcinomas of the cervix, head and neck [12]. DSBs produced by BL have blunt ends or 1-base 5′-overhangs. At the 3′-ends, deoxyribose sugar moiety is oxidized at the C-4′ position that leads to 3′-phosphoglycolate (PG) formation [13]. For repair of DSBs containing 3′-PG termini, end processing is required.

DSBs are especially dangerous for cells because they inhibit transcription and replication [14, 15], and lead to genomic rearrangements and the appearance of chromosome aberrations. DSBs are repaired by non-homologous end-joining (NHEJ) or homologous recombinational repair (HR). NHEJ is considered to be the main pathway of DSB repair that occurs during all phases of the cell cycle, but is predominant in G0/G1 [16], while HR is absent in G1, the most active in S and G2, and decreases when cells progress to G2/M stage [17]. DNA-PK, DNA-ligase IV, XRCC4, XLF, PNKP, Tdp1, Artemis and DNA-polymerases µ and λ operate in NHEJ [13, 16, 18, 19]. HR begins with the recognition of DSB by Mre11/Rad50/NBS1 (MRN complex) followed by resection of broken DNA ends by MRN together with CtIP. Generated 3′ DNA ends are covered by RPA, which is replaced by Rad51, and Rad51-formed filaments invade homologous sequence [20].

The induction of the phosphorylated form of histone H2AX, called gamma-H2AX (gH2AX), is one of the earliest events involved in DDR. gH2AX induction is a crucial event in DSB repair that leads to the recruitment of a number of other repair proteins at the sites of DSBs [21, 22]. H2AX phosphorylation could be detected by Western blotting or immunostaining in combination with fluorescence microscopy. DSB sites can be easily visualized in cell nuclei as local spots of H2AX histone phosphorylation. It has been shown that the number of DSBs corresponds to the number of gH2AX foci in cell nuclei. Approximately the same number of DSBs, 35 per Gy per cell, is induced in different cells treated by IR [23]. The immunofluorescence detection of gH2AX is considered as the most sensitive method of recognition of DSB sites in cell nuclei.

Using these approaches, we studied the effectiveness of BL-induced DSB repair in young and presenescent Syrian hamster fibroblasts and the kinetics of recruitment of phospho-(Ser1981) ATM (pATM), 53BP1 and phospho-(Ser2056) DNA-PK (pDNA-PK) DSB repair proteins to DSB sites marked by gH2AX. Using immunoblotting technique, we could not find any difference in kinetics of gH2AX loss during 24 h after BL treatment of cells at the 1st and the 5th passages. Nevertheless, we observed some differences in DDR between young and presenescent Syrian hamster cells using immunofluorescence microscopy technique. The heterogeneity of the number of DSBs per cell characterized both presenescent and young fibroblasts at different stages of the cell cycle. At the 1st passage, the average number of BL-induced DSBs per nucleus in proliferating G1 cells was similar to that in G0 cells. The gradual decrease in the average number of foci per nucleus in G0 cells at early passage was more pronounced than that in cells at late passage. Differences in kinetics of pDNA-PK, 53BP1 and pATM recruitment to DSB sites were observed in young fibroblasts in comparison to non-proliferating cells at late passage.

## Results

### Changes in morphology and β-galactosidase activity in Syrian hamster fibroblasts

Syrian hamster fibroblasts obtained from newborn animals stopped proliferation after 5 passages, 7 population doublings (PD 7) in standard conditions of cultivation and displayed morphological changes typical for cellular senescence, i.e. enlarged nuclei and flattened cytoplasm, that differed significantly from spindle-shaped cytoplasm of young cells (Additional file 1: Figure S1, Additional file 2: Figure S2).

In human cells, a senescent phenotype usually correlates with the appearance of β-galactosidase (β-gal) activity in the cytoplasm after application of a β-gal assay at pH6 [24, 25]. The increase of β-gal activity in Syrian hamster fibroblasts that prematurely stopped dividing was not observed in this study (Additional file 1: Figure S1). At the 38th passage, practically all replicatively senescent human VH-10 fibroblasts displayed a dense blue color after application of β-gal assay due to a high level of enzyme expression. Syrian hamster cells stopped dividing at the 5th passage in conventional conditions of cultivation, while only some of them showed a faint blue staining.

β-gal-positive cells were observed in cultures of senescent human and rat fibroblasts, but there was no β-gal activity in senescent mouse cells [26]. These data likely indicate that β-gal expression cannot be considered as a universal marker of cell senescence. However, non-dividing β-gal-negative Syrian hamster fibroblasts grown in conventional conditions of cultivation do not formally fall into the category of classical β-gal-positive senescent cells and were, therefore, determined here as cells in a presenescent state.

### Kinetics of gH2AX formation and elimination in BL-treated cells estimated by immunoblotting

**Fig. 1:**
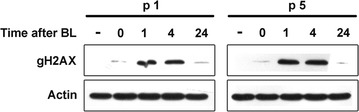
Kinetics of gH2AX formation and elimination in young and senescent Syrian hamster fibroblasts treated with BL. The cells at different passages were treated with 50 µg/ml BL for 30 min, washed with PBS and incubated in BL-free medium for the period of time indicated in the figure. Cell extracts were resolved by SDS-PAGE and analyzed by Western blotting using antibodies specific to gH2AX and beta actin

Using a Western blot technique, the total level of gH2AX phosphorylation was compared in young and presenescent Syrian hamster fibroblasts. Untreated cells and cells after BL-treatment (30 min) were incubated in BL-free culture medium for 1, 4 and 24 h (Fig. 1).

In cells of early and late passages, gH2AX content was increased at 1 h after the treatment, no detectable gH2AX loss was observed at 4 h, and a significant decrease in gH2AX content was registered at 24 h post treatment.

### Heterogeneity of the number of BL-induced DSBs in Syrian hamster fibroblasts

The kinetics of gH2AX formation and elimination evaluated by Western blotting technique reflects only alterations of the total gH2AX level in cell nuclei. For individual nuclei, the counting of gH2AX foci is an appropriate method for monitoring DSB repair. In the present study, this approach was used for enumeration of gH2AX foci in BL-treated Syrian hamster cells at different stages of the cell cycle.

**Fig. 2:**
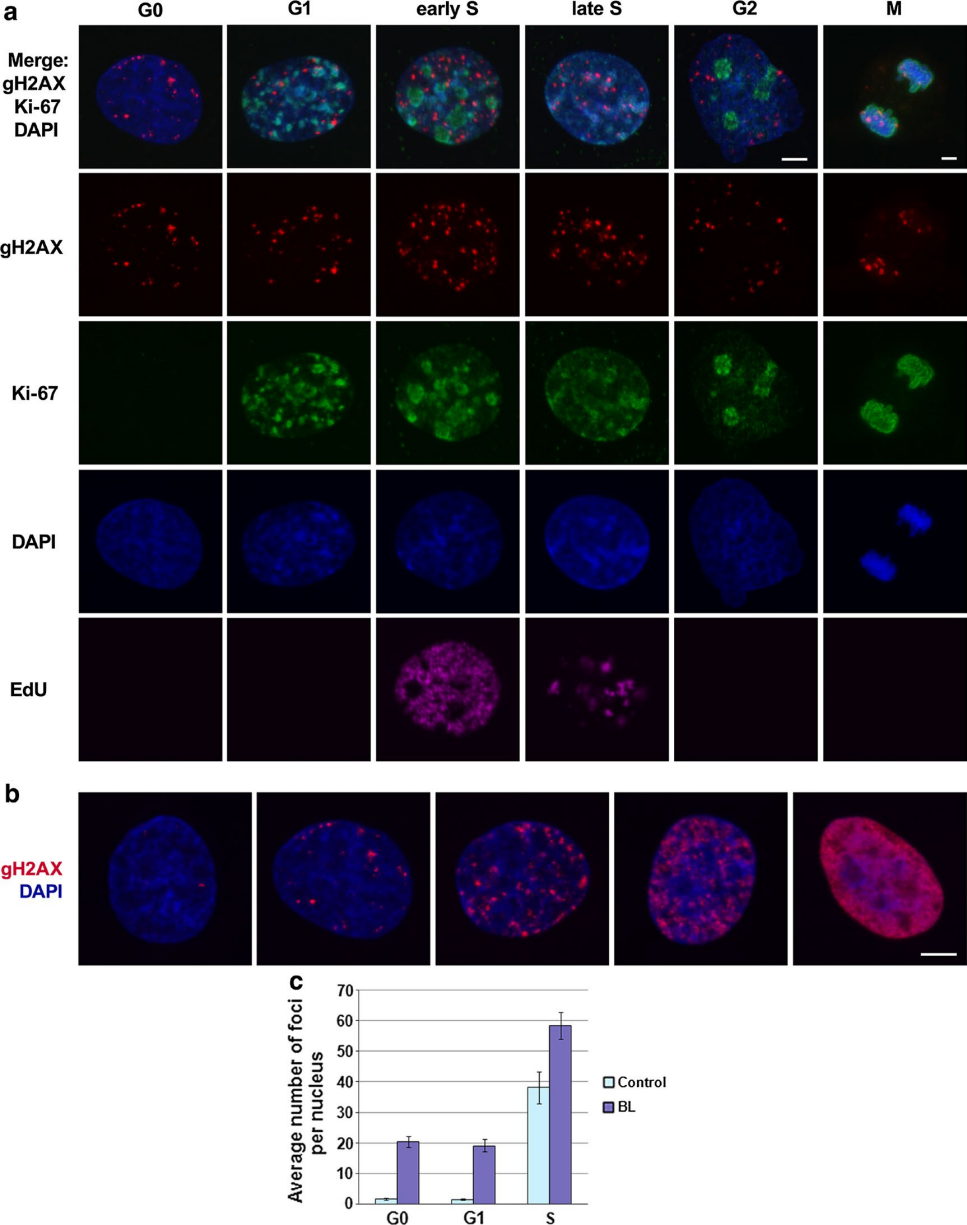
Visualization of gH2AX foci and variation in the number of foci per cell in Syrian hamster fibroblasts. **a** Visualization of gH2AX foci in nuclei of cells at different stages of the cell cycle determined by Ki-67 staining (early-passage cells are shown). *Bars* are 5 µm (shown in G2 cell image for all interphase cells, and in M—for anaphase cell). **b** Examples of BL-treated (0 h after the treatment) G0 cells at the 1st passage with variable countable numbers of gH2AX foci and a cell containing multiple breakage sites with a uniform staining with the use of anti-gH2AX antibody. *Bar* is 5 µm. In **a** and **b**, maximal projections of confocal sections are shown. In late-passage cells, similar types of gH2AX focus distribution were observed as in G0 early-passage cells. DNA in **a** and **b** is counterstained with DAPI. **c** *Bar chart* representing average numbers of foci per cell nucleus in control and BL-treated G0, G1 and S-phase cells at the 1st passage collected at 0 h after BL treatment. Around 200 cells were analyzed for each phase of the cell cycle. *Error bars* represent ±3 SE

For determination of certain phases of the cell cycle, double gH2AX/Ki-67 immunostaining combined with detection of 5-ethynyl-2’-deoxyuridine (EdU) incorporated in S-phases was performed on Syrian hamster fibroblasts treated with BL. Ki-67 localization in the nuclei is cell cycle-dependent, and characteristic patterns of Ki-67 nuclear distribution in Syrian hamster fibroblasts, human fibroblasts and ES cells were described earlier [27–29]. In all cell lines, Ki-67 staining was visible only in proliferating cells, and G0 cells were Ki-67 negative. Syrian hamster G1 cells were characterized by a low level of nucleoplasm staining and bright staining of multiple loose nucleoli. Ki-67 distribution in early S-phase was very similar to that in G1, therefore it was difficult to discriminate early S-phase from G1 without using additional EdU incorporation (Fig. 2a).

Individual BL-treated Syrian hamster cells contained variable numbers of gH2AX foci at any stage of the cell cycle. A great variation in the number of gH2AX foci per cell, from low to very high, characterized BL-treated Syrian hamster cells, both young G0 and presenescent (Fig. 2b). This phenomenon was not dependent on the level of BL-hydrolase (BLH) expression, an enzyme that catalyzes inactivation of BL. BLH foci were located in the cytoplasm of Syrian hamster cells, and the density of BLH immunostaining was similar in cells having different numbers of gH2AX foci (Additional file 3: Figure S3).

G1 and G0 Syrian hamster cells had similar susceptibility to BL damage: at the 1st passage, immediately after 30 min-BL treatment, average numbers of gH2AX foci per cell were equal in both phases of the cell cycle (Fig. 2c).

Among untreated S-phase Syrian hamster cells of the 1st passage, a large variation in the number of gH2AX foci per cell was observed, and it was approximately 1.5 times greater after BL treatment (Fig. 2c). It has been shown that formation of gH2AX foci in S-phase cells occurs after replication stalling and is ATR-kinase dependent [30]. gH2AX foci could be induced as a result of DNA replication fork stalling at the sites of oxidative damage (for example, 8-oxoguanine) accumulated during cultivation [31].

The number of gH2AX foci per cell was also variable in G2 phase of the cell cycle in Syrian hamster cells (an average number of foci per cell is not shown due to a limited number of G2 cells in culture).

At late passages, the enumeration of gH2AX foci in cells at different phases of the cell cycle was not performed since very few proliferating cells were observed in the culture.

### Observation of BL-treated cells with dense gH2AX staining

DDR in eukaryotic cells results either in cell cycle arrest, to allow the lesions to be repaired, or in apoptosis. Apoptotic cells are known to undergo DNA fragmentation due to the action of nucleases, and gH2AX formation is an early chromatin modification following the initiation of DNA fragmentation [32]. It has been shown that BL can initiate a death-receptor-dependent, extrinsic apoptosis pathway [33].

**Fig. 3:**
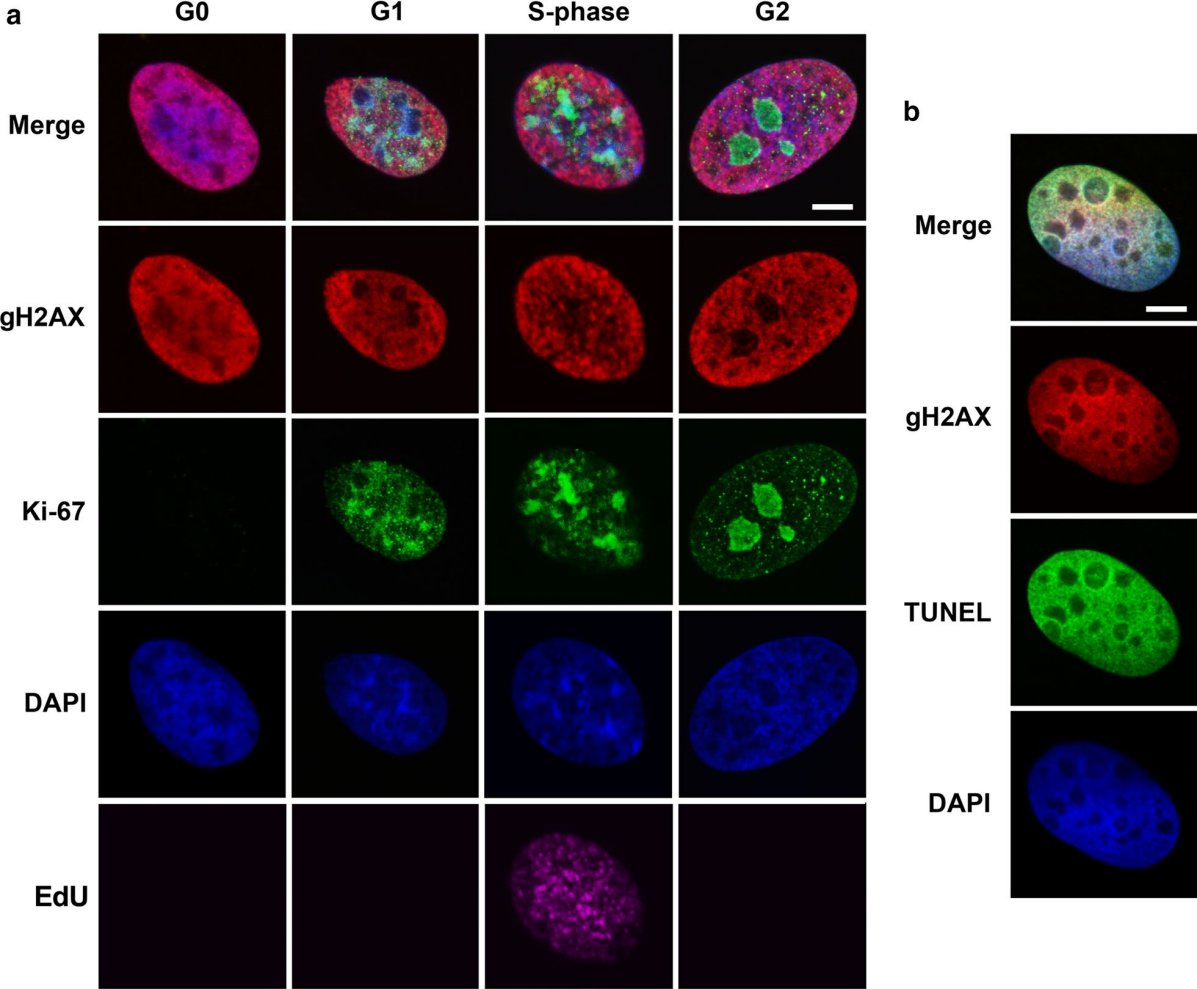
Transition of Syrian hamster cells from different stages of the cell cycle into apoptosis. **a** Nuclei of BL-treated cells (0 h after BL-treatment) at different stages of the cell cycle (determined by Ki-67 staining and EdU incorporation) with dense gH2AX staining are shown (*left panel*). **b** TUNEL assay (*right panel*) confirmed that cells densely stained with anti-gH2AX antibody transited into apoptosis. Maximal projections of confocal sections (*left panel*) and a single confocal section (*right panel*) are shown. *Bar* is 5 µm

We observed that a fraction of BL-treated cells were homogenously stained with the use of anti-gH2AX antibody. This type of dense staining was detected in cells at all stages of interphase. These cells were positively stained in the TUNEL (terminal deoxynucleotidyl transferase dUTP nick end labeling) assay, i.e. represented apoptotic cells. Interestingly, at both the 1st and the 5th passages, the cells homogenously stained for gH2AX could be detected immediately after 30 min of BL treatment (Fig. 3).

### Kinetics of DSB focus loss in BL-treated G0 young and presenescent fibroblasts

**Fig. 4:**
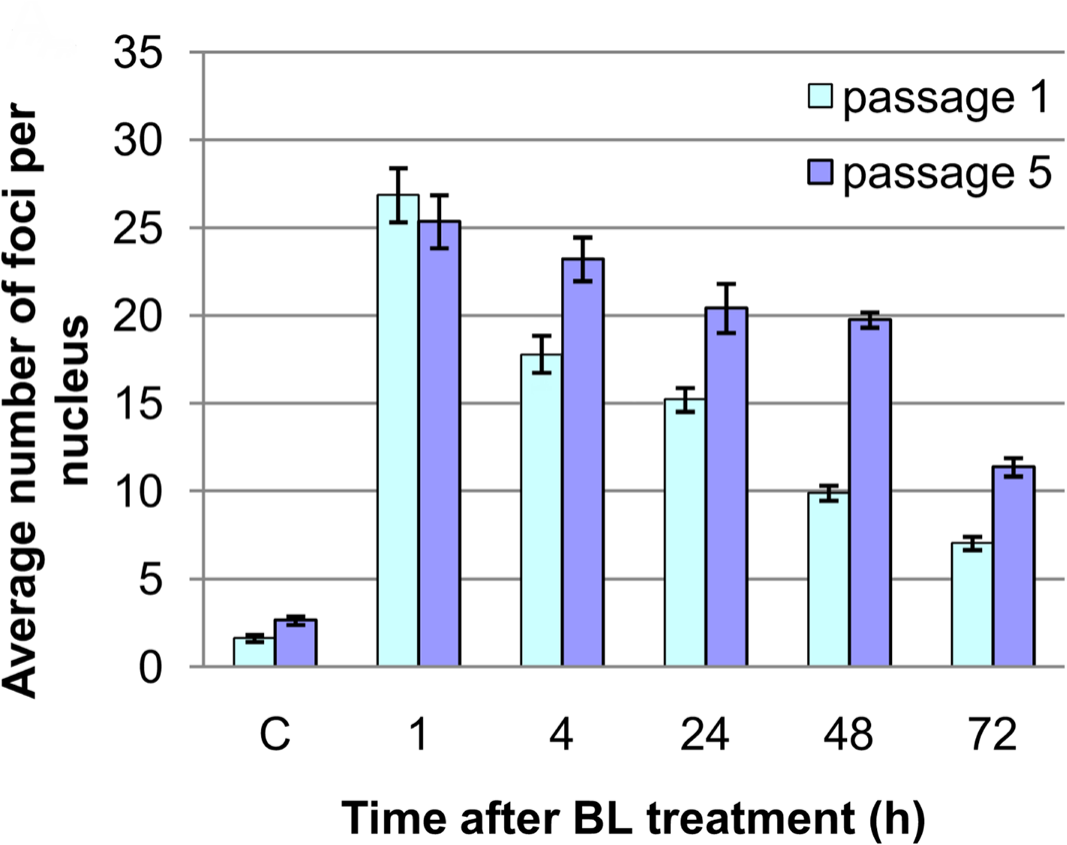
gH2AX focus formation and loss in BL-treated Syrian hamster fibroblasts. *Bar chart* representing gH2AX focus counts per nucleus in G0 fibroblasts of early (*passage 1*) and late (*passage 5*) passages. 200 cells were analyzed for each time point, and the average numbers of foci per nucleus ±SE are shown. The differences between average numbers of foci per nucleus in early- and late-passage cells are statistically significant (p < 0.01) for the time points 4, 24, 48, 72 h. “C” indicates the charts for gH2AX focus counts in non-treated (control) cells

We compared the kinetics of gH2AX focus loss in presenescent cells at the 5th passage and non-dividing G0 cells at the 1st passage. Cells containing countable numbers of foci were taken for analysis, and cells with a homogenous type of gH2AX staining were omitted. Approximately the same numbers of gH2AX foci were induced in young and presenescent cells 1 h after the treatment (Fig. 4). A difference in gH2AX focus elimination between G0 young and presenescent cells was detectable 4 h after the treatment: 33.4 % of foci were repaired in young cells versus 8.4 % in presenescent ones. A bigger difference in the fraction of repaired DSBs was observed 48 h post treatment, when 63 % of foci were lost in young cells and only 15 % in presenescent ones. A significant gH2AX focus loss in presenescent cells was observed only 72 h after the treatment (Fig. 4).

We also estimated the kinetics of gH2AX focus size changes in Syrian hamster fibroblasts during 3 days post BL treatment. Histograms of the distribution of projected area of gH2AX foci (PAF) in nuclei of young and presenescent cells were created (Additional file 4: Figure S4). A wide distribution of focus sizes was observed in cells of early and late passages (from 0.24 µm^2^ to more than 5.0 µm^2^). For both passages, histograms of PAF have a skewed-right distribution in the direction of larger PAF values. In histograms representing PAFs in cells of both passages 1 h after BL treatment, peak values corresponded to 0.4–0.49 µm^2^. An overall tendency in gH2AX focus size increase was observed both in young and presenescent cells during 3 days after BL treatment. A time-dependent decrease of peak fraction of PAFs was registered in both cultures, young and presenescent. This decrease was more pronounced in young Syrian hamster cells: 2 days after BL treatment, this fraction of PAFs decreased significantly in cells of the 1st passage, while, only in 3 days, some decrease of this fraction was observed in presenescent cells.

In untreated Syrian hamster fibroblasts, about 50 % of cells in cell culture of the 1st passage and 60 % of cells in presenescent culture contained gH2AX foci. An average PAF in untreated cells corresponded to 0.81 µm^2^ at the 1st passage and to 0.84 µm^2^ at the 5th passage. Foci of different sizes in the range of 0.24–5.00 µm^2^ could be observed in the same nucleus. In contrast, in a confluent human early-passage fibroblast culture, two fractions of cells were observed: cells containing only small foci (0.7 µm^2^) and cells with large foci (1.7 µm^2^) [34].

### Distribution of pATM, pDNA-PK and 53BP1 in interphase and mitotic BL-treated Syrian hamster cells

ATM, DNA-PK and 53BP1 are involved in main DSB repair pathways in mammalian cells and are accumulated specifically at DSBs marked by gH2AX in damaged chromatin of interphase cells. The distribution of phospho-(Ser1981) ATM, phospho-(Ser2056) DNA-PK and 53BP1in mitotic Syrian hamster cells was studied 24 h after BL treatment.

**Fig. 5:**
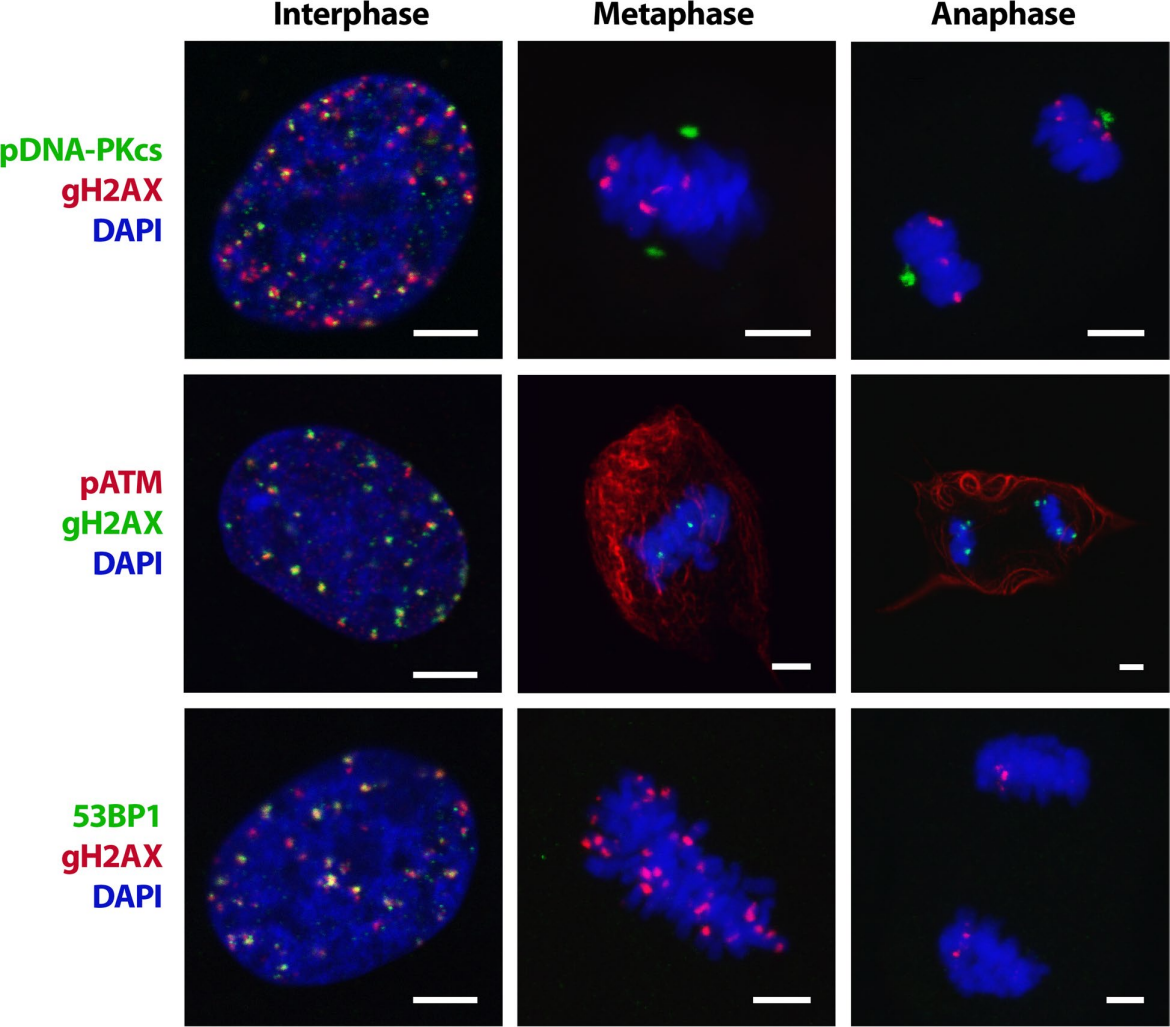
Distribution of gH2AX, pDNA-PK, pATM and 53BP1 in interphase and mitotic Syrian hamster cells. Confocal images of the 1st-passage cells collected 1 h after BL treatment are represented. *Colors* of immunofluorescent staining of proteins (*green or red*) are indicated on the *left*. DNA in cell nuclei is counterstained with DAPI (*blue*). Maximal projections of confocal sections are shown. *Bars* are 5 µm

It was previously reported that most of checkpoint-proficient cells were arrested at G2/M after IR, but a few cells could pass mitosis with 10–20 unrepaired gH2AX foci [35]. In the present study, we demonstrated that some mitotic Syrian hamster cells pretreated with BL contained a number of gH2AX foci (Fig. 5). It is interesting to note that, at the opposite poles of the spindle, anaphase chromosomes often contained a similar number of sites of gH2AX accumulation.

The evidence has been presented that DNA-PK, ATM and 53BP1 are involved not only in DDR but also play a role in mitotic checkpoint signaling. It has been shown that, in HeLa and mouse cells, 53BP1 is loaded onto kinetochores in prophase and is released after metaphase-anaphase transition [36]. In contrast, we were unable to detect 53BP1 foci in Syrian hamster metaphase chromosomes (Fig. 5) that was in agreement with the data obtained on MRC-5 human foetal lung fibroblasts [37].

Inactivation of DNA-PK results in multiple mitotic progression disorders, especially in abnormal chromosome segregation and failure of cytokinesis. Different phosphorylated forms of enzyme likely have distinct functions during the cell cycle. It has been reported that in HeLa cells, DNA-PK phosphorylated at Thr2609 colocalizes with centrosomes and kinetochores from prophase to anaphase and is accumulated in the midbody during cytokinesis, while DNA-PK phosphorylated at Thr2647 and Ser2056 is accumulated only in centrosomes [38]. In response to IR-induced DSBs, DNA-PK is phosphorylated at Thr2609 and Ser2056 in interphase cells [39]. Using double immunostaining with antibodies to gH2AX and phospho-(Ser2056) DNA-PKcs, we have found that gH2AX foci colocalize with phospho-(Ser2056) DNA-PK in interphase Syrian hamster cells, but these proteins do not exhibit colocalization in mitosis, where pDNA-PK is concentrated only in centrosomes.

It has been shown that ATM activity is enhanced in mitosis, and phospho-(Ser1981) ATM colocalizes with centrosomes at all stages of mitosis in human lymphoblastoid cells [40]. Using antibodies to phospho-(Ser1981) ATM, we did not detect a specific staining of centrosomes in Syrian hamster mitotic fibroblasts; instead, we observed a bright staining of a filamentous network in the cytoplasm outside the spindle (Fig. 5).

Our observations show that gH2AX foci do not colocalize with 53BP1, pDNA-PK and pATM in mitotic Syrian hamster cells indicating that H2AX phosphorylation is not associated with DSB repair in mitosis.

### Differences in the rates of pDNA-PK, 53BP1 and pATM recruitment to DSB sites in interphase nuclei

The recruitment of 53BP1, pDNA-PK, pATM and phospho-ATM/ATR substrate proteins (pSub) to DNA sites marked by gH2AX was estimated on Syrian hamster cells at early and late passages at two time points after BL action: immediately after the end of incubation with BL (0 h) and 1 h after the treatment. For exclusion of S-phase cells from the analysis, EdU incorporation was used as a marker of DNA replication synthesis. Quantitative colocalization analysis was applied to validate the overlay of signals of the proteins in green and red channels. The mean Pearson’s coefficient (Rr) and the mean Manders’ overlap coefficient (R) were calculated to estimate the degree of colocalization.

The coefficients of colocalization averaged for 25 cells at two time points after BL treatment were compared for the 1st and the 5th passages separately (Additional file 5: Table S1). At the 1st passage, the degree of colocalization of gH2AX with 53BP1, pDNA-PK and pATM did not differ at 0 and 1 h after BL. At the 5th passage, the degree of colocalization of gH2AX with 53BP1 and pDNA-PK proteins was higher at the 1 h time point when compared with the 0 h time point, but there was no increase in the rate of pATM recruitment during this period of observation.

**Fig. 6:**
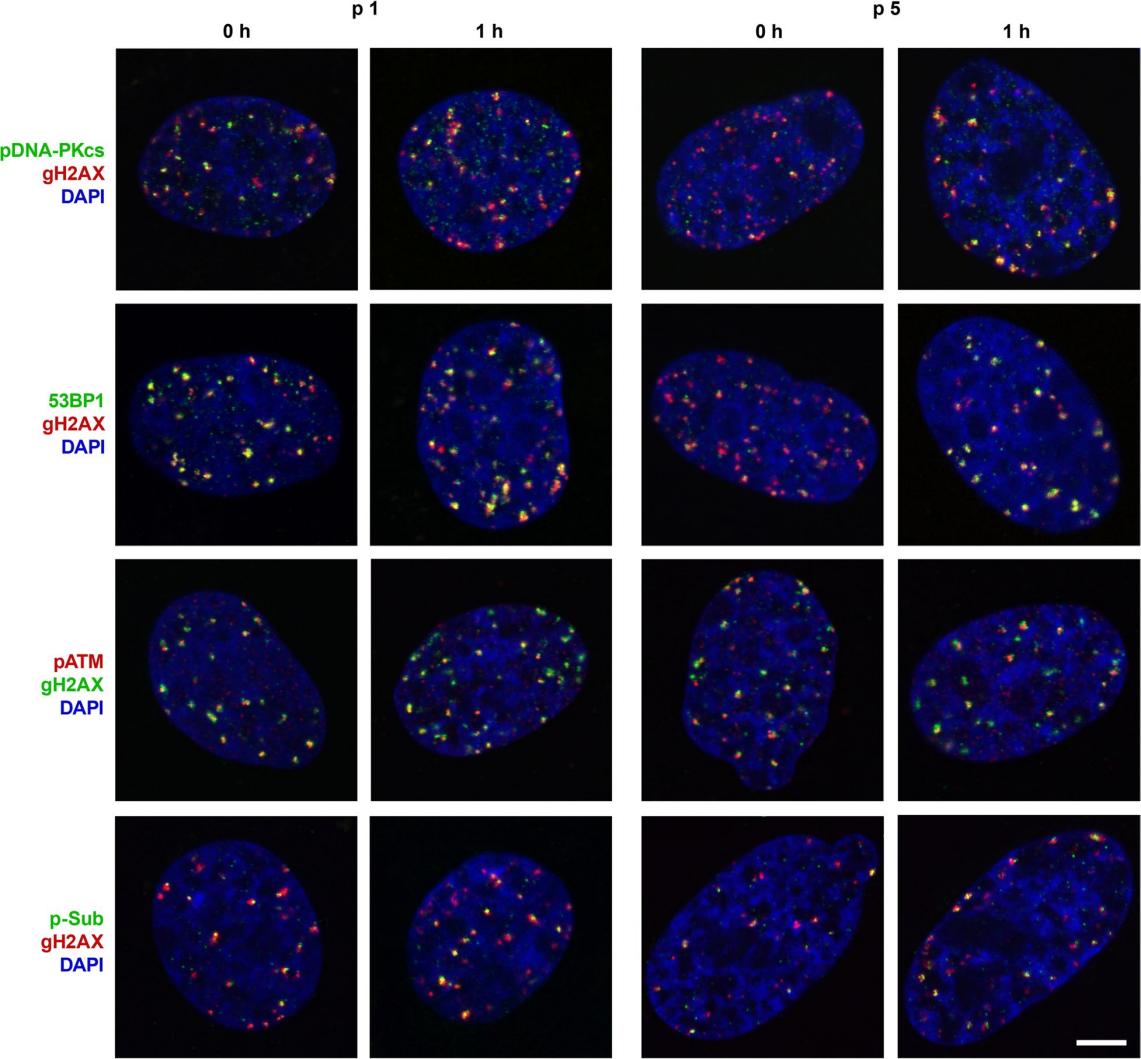
Localization of pDNA-PK, pATM, 53BP1, ATM/ATR phosphorylated substrate proteins (p-Sub) and DSB sites marked by gH2AX. The examples of single confocal sections of cell nuclei of young (the 1st passage, p 1) and presenescent (the 5th passage, p 5) Syrian hamster fibroblasts at 0 and 1 h after BL treatment used for measurements of the degree of colocalization of indicated proteins with gH2AX sites are presented. *Bar* is 5 µm

When comparing Syrian hamster cells at early and late passages at 0 h after BL treatment, a statistically significant decrease of gH2AX and 53BP1 focus overlap in cells of the 5th passage was observed (probability value p < 0.01) (Fig. 6; Table 1). At 1 h after BL treatment, the difference in colocalization of gH2AX and 53BP1 was found to be nearing significance. It is interesting to note that we also observed a statistically significant retardation in 53BP1 recruitment to the sites of damage in replicatively senescent human VH-10 fibroblasts (passage 38) in comparison with cells of a younger culture (passage 19) (Table 1).

**Table 1:** Colocalization of gH2AX with DSB repair proteins in human and Syrian hamster fibroblasts

Protein colocalized with gH2AX	Time after BL (h)	Cell line/passage	Rr ± StErr	P value	R ± StErr	P value
53BP1	0	VH-10/19P	0.79 ± 0.02	*<0.001*	0.84 ± 0.01	*<0.001*
		VH-10/38P	0.45 ± 0.02		0.57 ± 0.02	
53BP1	0	SHF/1P	0.64 ± 0.02	*<0.001*	0.72 ± 0.01	*<0.001*
		SHF/5P	0.46 ± 0.03		0.57 ± 0.02	
53BP1	1	SHF/1P	0.68 ± 0.02	0.051	0.75 ± 0.01	0.118
		SHF/5P	0.72 ± 0.01		0.77 ± 0.01	
pDNA-PK	0	SHF/1P	0.51 ± 0.01	*<0.001*	0.60 ± 0.01	*<0.001*
		SHF/5P	0.35 ± 0.02		0.48 ± 0.01	
pDNA-PK	1	SHF/1P	0.47 ± 0.02	0.546	0.58 ± 0.02	0.283
		SHF/5P	0.45 ± 0.02		0.56 ± 0.02	
pATM	0	SHF/1P	0.65 ± 0.01	*0.004*	0.72 ± 0.01	*0.002*
		SHF/5P	0.59 ± 0.01		0.67 ± 0.01	
pATM	1	SHF/1P	0.64 ± 0.01	*<0.001*	0.71 ± 0.01	*<0.001*
		SHF/5P	0.54 ± 0.01		0.65 ± 0.06	
pSub	0	SHF/1P	0.48 ± 0.02	0.690	0.54 ± 0.02	0.770
		SHF/5P	0.46 ± 0.03		0.50 ± 0.03	
pSub	1	SHF/1P	0.54 ± 0.02	0.119	0.60 ± 0.01	0.102
		SHF/5P	0.49 ± 0.02		0.56 ± 0.02	

Colocalization analysis was performed for G0 and G1 EdU-negative cells

Rr—Pearson’s colocalization coefficient, R—Manders’ colocalization coefficient. In each variant, the coefficients are presented as mean ± standard error for 25 cell nuclei. Student’s *t*-test was used to compare values of coefficients obtained for the 1st and the 5th passages of Syrian hamster cells or the 19th and the 38th passages of human fibroblasts. Probability p < 0.05 (indicated in italics) means that the difference between values is statistically significant. Rr reflects correlation of pixel intensities in both channels, and the values of Rr close to 1 indicate a reliable colocalization. While the values of Rr range from 1 to −1, value of 0 for R coefficients means no colocalization, value of 1 means perfect colocalization

*VH-10* human primary fibroblasts, *SHF* Syrian hamster primary fibroblasts, *pSub* phospho-(Ser/Thr) ATM/ATR substrate proteins

The kinetics of pDNA-PK recruitment to DNA sites marked by gH2AX was similar to that of 53BP1: it was slower at 0 h after BL treatment of Syrian hamster cells of the 5th passage in comparison with young cells (p < 0.01). 1 h later, pDNA-PK recruitment was equal in young cells and cells of late passage (Fig. 6; Table 1).

A statistically significant decrease of gH2AX and pATM colocalization (p < 0.05 at 0 h and p < 0.01 at 1 h after BL) was observed at the 5th passage when compared with the 1st passage at both time points after BL indicating a stronger defect in pATM accumulation at DNA damage sites in comparison with 53BP1 and pDNA-PK (Table 1).

Thus, our studies have shown that Syrian hamster cells of late passage display the retardation of 53BP1, pDNA-PK and pATM accumulation at the sites of BL-induced DSBs that is in accordance with the conception that some steps of DDR are impaired in prematurely aged cells grown in conventional conditions of cultivation.

PI3-related kinases ATM and ATR phosphorylate a number of cell cycle and DNA repair proteins, including p53, p95/NBS1, MDM2, Chk2, BRCA1, CtIP and Chk1, that have S/TQ consensus phosphorylation motif [41]. It has been shown that untreated MRC5 fibroblasts of late passage undergoing telomere-initiated senescence display colocalization of phospho-S/TQ and gH2AX foci [42].

Using antibodies to ATM/ATR phosphorylated consensus target sequence, we have observed a tendency towards increasing colocalization of gH2AX with pSub in Syrian hamster cells at the 1st passage during a selected period of time from 0 h to 1 h after the treatment, while, at the 5th passage, there was no statistically significant difference in colocalization during the same time period (Additional file 5: Table S1). There was also no difference in colocalization of gH2AX and pSub foci when comparing cells at the 1st passage with cells at the 5th passage at both time points selected (Fig. 6; Table 1).

## Discussion

BL is a well-known radiomimetic antibiotic that has a long history of application in anti-cancer therapy. The study of BL action on tumor cells was begun more than two decades ago. Treatment with chemotherapeutic agents that induce DSBs in DNA may be toxic both for tumor and normal cells. Among other factors, the degree of toxicity of an agent may depend on the capability of cells to accomplish DSB repair. The effectivity of DSB repair in normal primary young and presenescent Syrian hamster fibroblasts after BL treatment was compared in this study.

Over the last 15 years, gH2AX accumulation at the sites of DSBs was widely used as a biological marker of this type of damage. Immunostaining with anti-gH2AX antibody has an advantage over other techniques because it allows the direct count of the number of DSBs in individual cells. In this study, using immunofluorescence microscopy, we show that the number of BL-induced gH2AX foci varies significantly between individual Syrian hamster fibroblasts, both in young cells and cells of late passages. The selective manner of BL action was observed earlier by microelectrophoretic technique and comet assay [43, 44]. As we show here, the heterogeneity of the number of gH2AX foci in Syrian hamster cells does not depend on the expression of BLH. However, we cannot completely exclude a possibility that individual cells differ in the level of BLH activity, and this probable dissimilarity may contribute to varied sensitivity of cells to BL. It was demonstrated earlier that BLH knockdown markedly increased the sensitivity of HeLa cells to BL, and, in some tumor cell lines, the high level of BLH correlated with an increased resistance to BL [45]. These results indicate that BLH can modulate the action of BL, but it is not excluded that other cellular factors may also influence the level of BL sensitivity among cells in a distinct cell population. It was shown that BL was internalized into cells through receptor-mediated endocytosis [46], suggesting that different sensitivity of individual cells might be due to variations in membrane receptor pools. Recent reports have demonstrated that BL can penetrate through the membrane of human cells using l-carnitine transporter hCT2, and modulation of hCT2 expression levels could mediate the cellular resistance to this drug [12, 47].

In the present study, we analyze the kinetics of H2AX phosphorylation in Syrian hamster fibroblasts after BL treatment using immunoblotting and laser-scanning immunofluorescence confocal microscopy. We show by immunoblotting technique that the kinetics of DSB repair does not differ in early- and late-passage Syrian hamster cells. The level of gH2AX is similar at 1 and 4 h after BL, and a detectable loss of total gH2AX is observed 24 h post treatment. Using the same method of analysis, the maximal gH2AX induction was registered in cells of tumor cell lines and human lymphocytes 1 h after IR, and then the level of gH2AX slowly decreased [48, 49]. Immunoblotting technique allows the possibility to estimate only the total level of immunolabeled protein. However, H2AX phosphorylation is significantly increased in pre-apoptotic Syrian hamster cells, and the total level of gH2AX does not correctly reflect the input of focal gH2AX associated directly with DSB repair.

Confocal microscopy analysis performed in this study shows that the maximal number of gH2AX foci per cell in G0 Syrian hamster fibroblasts at the 1st passage is observed at 1 h after BL treatment. 4 h post treatment, the average number of gH2AX foci per cell is decreased to approximately 60 % of the maximal value. After that, it decreases at a slower rate and at 24 and 72 h corresponds to 57 and 26 % of the maximal value, respectively.

A similar DSB repair kinetics early (2–4 h) after BL treatment was observed by other researchers in primary asynchronous human skin fibroblasts and unstimulated lymphocytes [50, 51]. The mean tail moment in the comet assay performed using BL-treated cells of human lymphoblastoid TK6 cell line was decreased by approximately 50 % 3–4 h post treatment [52]. From 4 to 24 h post treatment, gH2AX elimination after BL was faster in asynchronous human fibroblasts in comparison to G0 Syrian hamster cells: at 24 h, an average number of foci was 5 % in human fibroblasts [50] and 57 % in Syrian hamster cells (this study). Thus, BL-induced DSB repair in G0 Syrian hamster fibroblasts at the 1st passage is slower than in human cells, indicating that, even in young Syrian hamster cells, the effectivity of DSB repair machinery is reduced in conventional conditions of cultivation. At the 5th passage, after BL, a statistically significant decrease in the rate of gH2AX focus elimination was observed at all time points (from 4 to 72 h) analyzed in comparison with the rate of elimination in cells at the 1st passage.

In G0/G1 mammalian cells, DSB repair is accomplished only by NHEJ, whereas HR pathway can take place exclusively after DNA replication and is important in S and G2 phases of the cell cycle [19]. Senescence-associated changes in NHEJ were found in normal human fibroblasts: the repair of DSBs introduced by a rare-cutting endonuclease was 4.5 times slower in old cells in comparison with that in young ones [53]. The frequency of precise ligation of DSB ends was higher in young cells, and the end-joining ability in senescent human cells and tissues of old mice was both inefficient and error-prone [54].

An alternative backup NHEJ pathway (B-NHEJ), that is mediated by PARP1, LigIII and histone H1, could substitute the canonical pathway of NHEJ (D-NHEJ) in cases when the latter becomes compromised [55–57]. It has been reported that D-NHEJ operates efficiently in all phases of the cell cycle and is reduced in plateau-phase cells in comparison with exponentially growing ones. B-NHEJ has been also shown to be growth-state dependent: it is markedly decreased in the plateau phase of D-NHEJ-deficient cells [58]. In this study, we demonstrate the impairment of gH2AX focus elimination in non-replicating presenescent Syrian hamster cells that utilize NHEJ pathway of DSB repair. It is a subject of further research to determine whether D-NHEJ may be substituted by alternative slow backup NHEJ machinery in presenescent and senescent cells.

gH2AX focus size analysis in BL-treated Syrian hamster cells has shown that distribution of projected area of foci (PAF) differs in young and presenescent cells after BL treatment. The peak fraction of small foci (0.40–0.49 µm^2^) gradually decreases during 3 days, disappears finally in young cells, but persists in cells of late passage.

We observed accumulation of large persistent gH2AX foci in Syrian hamster cells of both early and late passages. The long-time persistence (3 days) of small foci in late-passage cells might result from insufficiency of DSB repair machinery in conventional conditions of cultivation. gH2AX focus size was analyzed earlier in human fibroblasts within a short time (40 min) of incubation after IR: in comparison with young fibroblasts, a slower enlargement of gH2AX foci in cells at high population doubling was observed, indicating reduction of DSB repair efficiency in aged cells [59].

Conventional conditions of cultivation permanently induce oxidative damage in Syrian hamster cells that leads to prolonged DDR signaling. A variety of stresses, which result in DNA damage, could be considered as inducers of senescence [60, 61]. Senescence depends on activation of p53/p21 and p16 INK4a/RB signaling pathways [62, 63]. It has been shown that IR-induced DNA damage leads to formation of two categories of gH2AX foci: small transient repairable foci and large persistent irreparable foci that can be observed after a period of time sufficient to resolve transient foci [64, 65]. These stable foci are called DNA segments with chromatin alterations reinforcing senescence (DNA-SCARS) induced by DNA damage. Both types of foci accumulate DSB repair proteins, but persistent foci lack RPA and RAD51 proteins, single-stranded DNA, and DNA repair synthesis. DNA-SCARS maintain permanent DDR, lead to DNA damage-induced cell growth arrest and senescence. Persistent DNA damage signaling is associated with the appearance of SASP, which is associated with the secretion of pro-inflammatory cytokines and mediators of inflammatory reactions including interleukin-6, interleukin-8, insulin-like growth factor binding protein 7, matrix metalloproteinases and others [60, 61, 66, 67]. p38MAPK is a DDR-independent regulator of SASP [5].

Persistent DDR signaling in Syrian hamster cells grown in conventional conditions of cultivation is induced by permanent ROS-induced DNA damage. During cultivation, Syrian hamster cells acquire the features of SASP and stop dividing after several passages (PD 7). In conditions of chronic cultivation stress, the capability of DNA repair machinery to respond to BL-induced stress is decreased in late-passage cells. Thus, there is a tight link between the acquisition of senescence-like state and DSB repair capacity. We suggested that, among other reasons, inefficiency of DSB repair might be due to impaired recruitment of DSB repair proteins to DSB sites. Therefore, we analyzed distribution of 53BP1, pDNA-PK and pATM in interphase and mitotic cells.

We have shown that foci of DDR proteins, pATM, pDNA-PK and 53BP1, partially overlay with gH2AX foci in interphase Syrian hamster cells. The presence of gH2AX sites in mitotic chromosomes of untreated or irradiated human cells was previously reported [68, 69]. DNA damage signaling is attenuated in mitotic cells: gH2AX foci colocalize with Mre11 and NBS1, but not with 53BP1, pATM and pDNA-PK [69, 70]. In Syrian hamster mitotic cells, we did not observe gH2AX colocalization with 53BP1 after BL treatment similar to observations made by other researchers [37, 71]. 53BP1 dissociates from DSBs at G2/M boundary [36]. Mitotic kinases CDK1 and PLK1 phosphorylate 53BP1 to inhibit its recruitment to the ends of DSBs [72].

In contrast to 53BP1, pDNA-PK is required for the normal function of spindle assembly. Phosphorylated forms of DNA-PK at Ser2056 and Thr2609 are critical for DSB repair [73]. During mitosis, DNA-PK is phosphorylated at multiple Ser and Thr residues: besides Ser2056 and Thr2609, Ser3205, Thr2647 and Thr3950 are also phosphorylated [74]. The precise functional consequences of these phosphorylation events in mitosis are yet to be elucidated. phospho-(Thr2609) DNA-PK was detected at centrosomes and kinetochores in metaphase, and at the midbody in cytokinesis [38, 75], while phospho-(Thr3950) DNA-PK was located at centrosomes and the midbody, and phospho-(Ser3205) DNA-PK at the midbody only [74]. Using Western blot technique, the increase of DNA-PK phosphorylation at Ser2056 was detected in mitotic normal human fibroblasts [73]. In the present study, using immunofluorence microscopy, we observed phospho-(Ser2056) DNA-PK localization only at centrosomes in normal fibroblasts of Syrian hamster at 1 h after BL. It has been shown that DNA-PK depletion sensitized cells to IR-induced mitotic catastrophe, suggesting that the association of pDNA-PK with centrosomes and kinetochores is necessary for spindle stabilization and correct mitotic progression [75].

ATM is involved not only in DDR in interphase cells, but it is also necessary for correct spindle assembly checkpoint to prevent chromosome segregation errors [76]. ATM controls phosphorylation and activation of Bub1 protein involved in transition from metaphase to anaphase. An activation of spindle checkpoint is dependent upon ATM Ser1403 phosphorylation that is performed by Aurora-B kinase. Diffused nuclear localization of phospho-(Ser1403) ATM is observed from prophase to anaphase [77].

In Syrian hamster fibroblasts, we observed phospho-(Ser1981) ATM localization on a filamentous network surrounding mitotic spindle. Nuclear lamina is composed of intermediate filaments and is located on the inner surface of the nucleus in interphase cells. Nuclear architecture is reorganized during mitosis, and mitotic changes include nuclear lamina disassambly and nuclear envelope breakdown [78]. We speculate that phospho-(Ser1981) ATM binding to protein filaments during mitosis may be associated with the process of nuclear lamina realignment. Clarification of the role of ATM in mitotic cells requires further investigation.

Here, we present evidence that the rates of recruitment of 53BP1, phospho-(Ser2056) DNA-PK and pATM to gH2AX foci after BL treatment are slower in senescent than in young G0 Syrian hamster cells. As it was shown earlier, 10 min post IR, gH2AX and Mre11 protein foci colocalized almost completely in low-passage human fibroblasts, while a significant decrease of the level of colocalization was observed in senescent cells [59].

53BP1 protein is one of the important regulators of DSB repair pathway choice. HR requires extensive DNA end processing that produces 3′-strand overhangs necessary for this type of DSB repair. Mre11, a component of DSB recognition MRN protein complex, in cooperation with CtIP, produces an initial limited resection at the sites of DSBs, and, after that, an extensive resection is mediated by the exonuclease Exo1, the key enzyme of DSB end processing [79]. It is considered that the main 53BP1 function in NHEJ is the prevention of DSB ends from extensive degradation [80].

DNA-PK, together with Ku70/80, is an important component of NHEJ. DNA-PK is involved in IR-induced H2AX phosphorylation and is also necessary for gH2AX elimination in mouse tissues [81]. DSB ends are recognized by Ku70/80, a heterodimer that keeps the broken DNA ends together. DNA-PK stabilizes the Ku heterodimer at DSB ends, and the phosphorylation of the C-terminal end of DNA-PK plays an essential role in regulation of this association [82]. It was shown that the level of Ku70/80 was decreased two-fold in replicatively senescent human cells. Moreover, intracellular localization of Ku70/80 differed in young and senescent human embryonic fibroblasts. In young cells, Ku70/80 was detected both in the nucleus and in the cytoplasm; however, in senescent cells, it was present only in the nucleus. After irradiation, Ku70/80 disappeared from the cytoplasm and relocated to the nucleus in young cells, but its localization did not change in senescent cells [83].

ATM is a key regulator of signaling pathways that are triggered in interphase cells in response to DSB induction. The targets phosphorylated by ATM include MRN protein complex associated with recognition of DSBs, histone H2AX, transcription factor p53, 53BP1, mediator of DNA damage-checkpoint protein 1 (MDC1), Chk1 and Chk2 checkpoint kinases, and other proteins [84]. ATM plays an important role in initializing HR in S and G2 phases of the cell cycle [85, 86]. DSB repair is comprised of a fast and a slow component. ATM-independent fast component participates in repair of the majority of DSBs, and ATM-dependent slow component is responsible for repair of 10 % of IR-induced DSBs with damaged termini [87].

It is considered that proteins involved in HR and NHEJ DSB repair pathways could compete for DSBs, but the basis of pathway choice remains unknown [20]. This idea is supported by the observation that proteins involved in both pathways are concentrated at the ends of DSBs.

In BL-treated cells, we observed a large proportion of gH2AX foci colocalized with pATM, pDNA-PK and 53BP1. The difference of coefficients of colocalization of gH2AX foci with foci of these proteins is statistically significant in young and presenescent cells. There was no statistically significant difference in the colocalization of gH2AX foci and pSub foci in early- and late-passage Syrian hamster cells. Using antibodies that recognize common phospho-S/TQ-motif of pSub involved in DDR, we were unable to estimate the rate of recruitment of individual proteins, and we cannot exclude that it could differ in cells of different passages.

BL-induced DSBs have a complex structure and represent one of the types of DSBs that are observed after IR. 3′-ends of BL-induced DSBs are blocked by phosphoglycolates that can be excised by a number of enzymes including tyrosyl-DNA phosphodiesterase, Artemis and apurinic/apyrimidinic endonuclease Ape1 [52]. The proper sequence of the assembly of DSB repair machinery, which involves a large number of protein complexes, determines its accurate functioning. We suggest that the slow DDR protein accumulation at the sites of DSBs in presenescent Syrian hamster cells, in which only NHEJ is operating, may cause disregulation of DNA end processing. The accelerated rate of DSB end resection could contribute to an age-related decrease in NHEJ efficiency. However, the precise biological consequence of the delay in accumulation of 53BP1, pATM and pDNA-PK for DSB repair process in Syrian hamster cells at late passages remains unknown.

The evidence was obtained that accumulation of presenescent and senescent cells occurred not only during in vitro cultivation. An age-associated increase in the number of these cells was also observed in human and rodent normal tissues [88, 89]. Cells from Hutchinson-Gilford progeria syndrome patients prematurely displayed the features of senescence [90]. In this study, we observed a delay in 53BP1 protein accumulation at gH2AX sites both in Syrian hamster cells of late passage and in senescent primary human fibroblasts. We suggest that in human aged cells a similar impairment in recruitment of the other DSB repair proteins to the sites of damage could be detected. Further studies are needed to confirm this possibility for characterization of age-related peculiarities of DSB repair machinery in senescent human cells and tissues.

## Conclusions

The antibiotic BL that induces DSBs in DNA is clinically used as a chemotherapeutic agent to treat several types of cancer. Aging could be considered as a health risk factor during chemotherapy treatment. Therefore, it is important to estimate age-related changes in the effectiveness of DSB repair in normal cells after the drug’s action. We have used Syrian hamster fibroblasts prematurely aged at the 5th passage in standard culture conditions as a model of cells in presenescent state for the study of DSB repair peculiarities after BL treatment. At the 5th passage, Syrian hamster fibroblasts halted replication and displayed morphological features of senescent phenotype, but did not exhibit β-gal staining. We have shown that, in comparison with G0 cells of young culture, slower DSB repair kinetics characterized non-dividing BL-treated cells at late passage. The recruitment of pDNA-PK, 53BP1 and pATM repair proteins to the sites of DSBs is retarded in cells at late passage, demonstrating a dysregulation of DSB repair machinery. These data show that NHEJ pathway of BL-induced DSB repair operating in non-dividing presenescent cells becomes compromised.

## Methods

### Ethic statement

This study was carried out in strict accordance with the recommendations in the Guide for the Care and Use of Laboratory Animals of the National Institutes of Health, Washington D.C., 1996 (Russian version approved by the Russian National Bioethics Committee of the Russian Academy of Sciences published by National Academic Press, 1996). The housing and feeding of adult Syrian hamsters was directed by a qualified veterinarian. The protocol used for euthanasia of newborn animals (1 day of age) was approved by the Committee on the Ethics of Animal Experiments at the Institute of Cytology, Saint-Petersburg, Russia. Neonatal Syrian hamsters were killed by decapitation or cervical dislocation by a person fully trained in the appropriate procedures to minimize suffering.

### Isolation of skin fibroblasts

The primary fibroblast culture was obtained using enzymatic digestion method as follows. Pieces of dissected skin of newborn Syrian hamsters were washed twice with PBS by centrifugation for 3 min at 120×*g*, placed in 3 volumes of enzyme mixture (10 mg/ml collagenase IV from *Clostridium histoliticum*, 2.5 U/ml dispase, 0.0001 % Trypsin–EDTA in PBS containing Ca^2+^ and Mg^2+^) and incubated for 20 min at 37 °C with shaking. The pieces were then dissociated into separate cells by pipetting, and the cell suspension was washed twice with PBS by centrifugation at 270×*g* for 5 min. After that, the cells were resuspended in culture medium and placed in a CO_2_-incubator.

Primary culture of human fibroblasts VH-10 was obtained from the Russian Cell Culture Collection at the Institute of Cytology RAS.

### β-gal detection and imaging

Senescence β-Galactosidase Staining Kit (Cell Signaling, Cat N. 9860) was used for estimation of β-gal expression in human VH-10 and Syrian hamster fibroblasts. The assay was performed according to manufacturer’s recommendations. We obtained the best results using short-term (4 min) incubation in fixative solution provided in the kit.

### Bleomycin sulphate treatment, cell fixation, immunofluorescent staining

Fibroblasts were grown on square 18 × 18 mm glass coverslips placed in Petri dishes in MEM supplemented with 15 % FCS, 2 mM l-glutamine and 100 U/ml streptomycin/penicillin. Bleomycin sulphate (BL) (Invitrogen) was added for 30 min to culture medium to a final concentration of 50 µg/ml. After the treatment, cells were washed twice with PBS and incubated in fresh culture medium for the times indicated in the text.

The cells on coverslips were fixed with 4 % formaldehyde in PBS at +4 °C, rinsed in PBS, kept overnight in 70 % ethanol, washed in PBS, permeabilized by 0.5 % Triton X-100 for 15 min at RT with shaking, washed in PBS and incubated for 30 min in 1 % Blocking Reagent (Roche, Cat N. 1096176) in PBS with 0.02 % Tween 20.

All antibodies were diluted in 0.5 % Blocking Reagent (Roche) solution with 0.02 % Tween 20. Incubations were performed at 37 °C, and slides were washed between subsequent incubations by shaking for 30 min in PBS supplemented with 0.1 % Tween 20. Cells were incubated for 1 h with the following primary antibodies: rabbit polyclonal anti-gH2AX (abcam, 1:100), mouse monoclonal anti-gH2AX (Upstate, 1:200), rabbit polyclonal anti-53BP1 (1:200), rabbit anti-phospho DNA-PKcs (S2056) (abcam, 1:100), mouse monoclonal anti-phospho ATM (S1981) (abcam, 1:100), rabbit polyclonal anti-Ki-67 (abcam, 1:200), rabbit polyclonal anti-BLMH (bleomycin hydrolase) (Proteintech, 1:50). Cells were incubated at +4 °C overnight with rabbit anti-phospho-(Ser/Thr) ATM/ATR substrate antibody (Cell Signaling, 1:100).

The secondary antibodies—Alexa Fluor 568-conjugated polyclonal goat anti-rabbit IgG or Alexa Fluor 488-conjugated polyclonal goat anti-mouse antibodies (Invitrogen, 1:400)—were added for 40 min, then the coverslips were washed for 30 min. DNA was counterstained with 0.5 μg/ml 4′,6-diamidino-2-phenylindole (DAPI) in PBS and mounted in Citifluor antifade solution (Marivac).

### EdU incorporation and detection

EdU (5-ethynyl-2′-deoxyuridine), the component of Click–iT EdU Imaging Kit (Invitrogen, Cat N. C10337), was added for 30 min to growth medium (10 µM final concentration) simultaneously with BL. The cells were fixed in 4 % formaldehyde in PBS for 15 min at RT, and all further steps were performed according to manufacturer’s recommendations, i.e. the coverslips were rinsed 2 times with 3 % BSA solution in PBS, permeabilized with 0.5 % Triton X-100 in PBS for 20 min at RT, then washed with 3 % BSA in PBS and treated for 30 min in the darkness with reagents of Click-iT cocktail including Azide-Alexa Fluor 647 triethyl ammonium salt. After blocking with 1 % Blocking Reagent (Roche), cells were stained with anti-gH2AX antibodies combined with anti-53BP1, anti-pDNA-PK, or anti-pATM antibodies, and DNA was counterstained with DAPI (blue emission). Red, far-red, green and blue fluorescence were acquired sequentially to avoid fluorophore emission bleed-through artifacts. The following fluorophore combinations were used: gH2AX (red, Alexa Fluor 568), 53BP1/pDNA-PK (green, Alexa Fluor 488), EdU (far-red, Alexa Fluor 647) or gH2AX (green), pATM (red), EdU (far-red).

### Immunoblotting analysis of gH2AX

Cells grown in 35 mm-diameter Petri dishes were lysed in 80 μl of SDS-PAGE sample buffer and kept for 10 min at 96 °C. After centrifugation, 10 μl protein samples were separated by 15 % SDS-PAGE, and proteins were transferred to Hybond C membranes (Amersham). The membranes were blocked overnight with 5.0 % non-fat dry milk in PBS containing 0.1 % Tween 20 (PBST). All incubations with antibodies were performed in 1 % BSA in PBST, and between incubations membranes were washed for 30 min in PBST. Membranes were subsequently incubated for 2 h at RT with mouse monoclonal antibodies to phosphorylated H2AX (Upstate, 1:2000), for 1 h at RT with horseradish peroxidase-labeled goat anti-mouse IgG (ZyMax, 1:10,000) and goat polyclonal antibodies to beta-actin (abcam, 1:1000). Immunoblots were visualized using Immobilon Western Chemiluminescent HRP Substrate (Millipore, Cat N. WBKLS0050).

### Detection of apoptotic cells

For detection of apoptotic cells, Click-iT TUNEL Alexa Fluor 488 imaging assay (Invitrogen) was used according to manufacturer’s recommendations. As a positive control, DNAse I treatment was applied to generate DNA breaks. After application of Click-iT TUNEL kit, cells were immunostained using mouse monoclonal antibodies to gH2AX (Upstate, 1:100) for visualization of gH2AX foci.

### Microscopy and image acquisition

For image acquisition, the confocal Leica TCS SP5 system equipped with HCX PL APO 100×/1.4 and 40×/1.25 oil immersion objectives, 488 nm argon, 543 nm HeNe, 594 nm HeNe and 405 nm diode lasers and Leica LAS AF software was used. The series of confocal sections were collected with the step size 0.5 μm. In a single confocal section, voxel size was 0.253 × 0.253 × 0.503 µm (zoom 1.5) for 40×/1.25 objective, and 0.033 × 0.033 × 0.503 µm (zoom 4.5) or 0.151 × 0.151 × 0.503 µm (zoom 1.0) for 100/1.4 objective. The image size was 1024 × 1024 pixels. Objective 40×/1.25 was used to capture large fields of view for counting of focus number per cell. For all other confocal images, objective 100×/1.4 was used. The gain of the signal was adjusted to obtain a linear working range. The same microscope settings were used for acquisition of images in each series of experiments.

Phase-contrast images of cells with blue beta-gal staining were collected using Zeiss Axiovert 200 M microscope equipped with DFC 420 colour Leica CCD camera with image resolution 2592 × 1944 pix and Plan-Neofluar 20 × 0.50 phase contrast objective.

### Counting of gH2AX foci per cell, measurement of gH2AX focus area

For counting of gH2AX foci per cell, Z-stacks of confocal sections of the nuclei were collected with 40×/1.25 objective at different time points during 3 days post BL treatment, and maximal projections of the series were obtained.

For area measurements, 100×/1.4 objective was used. The size of maximal projection of Z-stack was 909 × 909 pixels, and pixel size was 0.285 × 0.285 µm (zoom 1). For analysis of the area occupied by gH2AX foci (red, Alexa Fluor 568 staining) and the number of foci per nucleus, the segmentation function in IPLab program (Scananalytics, Inc.) was used. Red channels of RGB images were converted to grayscale images. In each series of experiments, the same thresholds of segmentation were applied for all images to discriminate the foci from background level. The area of each nucleus was taken as a region of interest (ROI). Inside the ROI, the area and the number of individual foci were calculated in pixels by IPLab program.

Projected focus area was converted to µm^2^ (one pixel area corresponded to 0.081 µm^2^), and histograms of focus area distribution were created using SPSS Statistics 17.0 program. Foci with projected area <3 pixels (0.24 µm^2^) were considered as the noise and were excluded from analysis.

### Colocalization analysis

Images of central confocal sections of the nuclei were taken for colocalization analysis of gH2AX foci with 53BP1, pDNA-PK or pATM foci.

The analysis of colocalization was performed using ImageJ 1.43 program (NIH) on the series of RGB images of cells in the “blind” manner, i.e. by a person without knowledge of particular proteins imaged in red and green channels.

The Difference of Gaussians (DoG) approach was used to locally segment focal signals of the repair proteins in the images. The DoG procedure relies on finding difference between two blurred versions of the original image, which are obtained by convolving the original grayscale image with Gaussian kernels having differing standard deviations. The first standard deviation (smaller Gaussian radius) corresponds to the frequency of noise, and the second standard deviation (bigger Gaussian radius) is selected based on the characteristic (maximum) size of the objects of interest. Subtracting one image from the other results in the segmented image where noise and background pixels are removed.

All analyzed images had 30 × 30 nm pixel size. Resolution with both green and red emission was ~200 nm. Therefore, we assumed that all 1-pixel spikes in intensity represented random noise, and 1 pixel was chosen as the smaller Gaussian radius. The maximum size of focal signals was estimated as ~25–30 pixels.

DoG segmentation was performed separately in red and green channels by convolving an image with Gaussian kernel with the radius of 1 pixel and subtracting the image convolved with Gaussian kernel with the radius of 30 pixels. The segmented image was normalized, and pixels outside of the nucleus were set to zero using ROI based on blue channel (DAPI staining). Colocalization between the segmented images of red and green channels was analyzed using “Manders’ coefficients” plug-in.

To verify the effect of the segmentation procedure on the results of the colocalization measurements, the DoG procedure was repeated on exemplary cells in each dataset using 5-pixel increments of the bigger Gaussian radius starting with 10 pixels. As expected, all measured parameters reached plateau at the radius of 30 pixels and varied within 5 % for the radius values from 30 to 55 pixels.

### Statistical analysis

Statistical analysis of the data was performed using Statistica 6 and GraphPad Prism 6 software. Significance of difference in numbers of gH2AX foci in young and presenescent cells at each of several time points (4, 24, 48, 72 h) after bleomycin exposure was determined using Student’s *t *-test.

In the analysis of DSB repair protein recruitment to gH2AX foci, for each type of protein, the mean values of Pearson’s and Mander’s correlation coefficients were determined for 25 cells for each time point (0, 1 h) after the treatment. Significance of the differential colocalization of DSB repair protein foci with gH2AX foci in young and presenescent cells was confirmed using a parametrical Student’s *t*-test, after verification of the normal distribution of the sampled coefficient values by Shapiro–Wilk test. Differences were considered significant at *p* < 0.05.

## Additional files

10.1186/s12867-015-0046-4 Detection of β-galactosidase activity in Syrian hamster early- and late-passage cells and human fibroblasts of different age. Phase contrast images of VH-10 human fibroblasts at the 19th and the 38th passages and Syrian hamster fibroblasts at the 1st and the 5th passages are shown. β-galactosidase is detected in blue color. Blue color is clearly expressed in senescent human cells, and only faint blue staining is observed in some of Syrian hamster presenescent cells. Bar is 50 µm.
10.1186/s12867-015-0046-4 Proliferation is arrested in Syrian hamster fibroblasts at the 5th passage. EdU was incorporated for 30 min in untreated Syrian hamster cells at the 1st (p 1) and the 5th (p 5) passages. Proliferating cells at the 1st passage are Ki-67-positive, S-phase cells incorporate EdU, G0 cells are Ki-67- and EdU-negative. All cells at the 5th passage are Ki-67- and EdU-negative. DNA in cell nuclei is counterstained with DAPI. Bar is 10 µm.
10.1186/s12867-015-0046-4 BL-hydrolase staining in Syrian hamster cells containing different numbers of DSBs. 1 h after BL treatment, cells containing different numbers of gH2AX foci demonstrate no visible difference in the density of cytoplasm staining by anti-BL-hydrolase antibody. Cells with one (A) and more than 20 (B) gH2AX foci are shown. Bar is 10 µm.
10.1186/s12867-015-0046-4 Histograms of projected gH2AX focus area distribution at different time points after BL treatment. Projected gH2AX focus area in young (p 1) and presenescent (p 5) Syrian hamster fibroblasts was measured after segmentation of images of maximal projections of confocal sections. Segmentation was performed using IPLab (Scananalytics) software with the same level of segmentation for all images. X axis—projected focus area (µm^2^), y axis—count of foci. Incubation time (1 h, 4 h, 24 h, 48 h, 72 h) after BL treatment is indicated, “C” indicates non-treated (control) cells. Sa—average projected area for indicated time point. 200 cells were taken for area measurements for each of histograms (with the exception for control histograms where 400 cells were taken).
10.1186/s12867-015-0046-4 Colocalization of gH2AX with 53BP1, DNA-PK, pATM and p-ATM/ATR substrates in Syrian hamster fibroblasts at two time points after BL treatment.

## Abbreviations

ATM: ataxia-telangiectasia mutated
DNA-PK: DNA-dependent protein kinase
PI3: phosphatidylinositol 3-kinase
ATR: ataxia-telangietasia and Rad3-related protein
NHEJ: non-homologous end-joining
HR: homologous recombinational repair
BL: bleomycin
