# Supplementary figures and images for: DNA double-strand break repair is impaired in presenescent Syrian hamster fibroblasts
Source: BMC Mol Biol. 2015 Oct 12;16:18. doi: 10.1186/s12867-015-0046-4 (PMC4601148; doi:10.1186/s12867-015-0046-4)

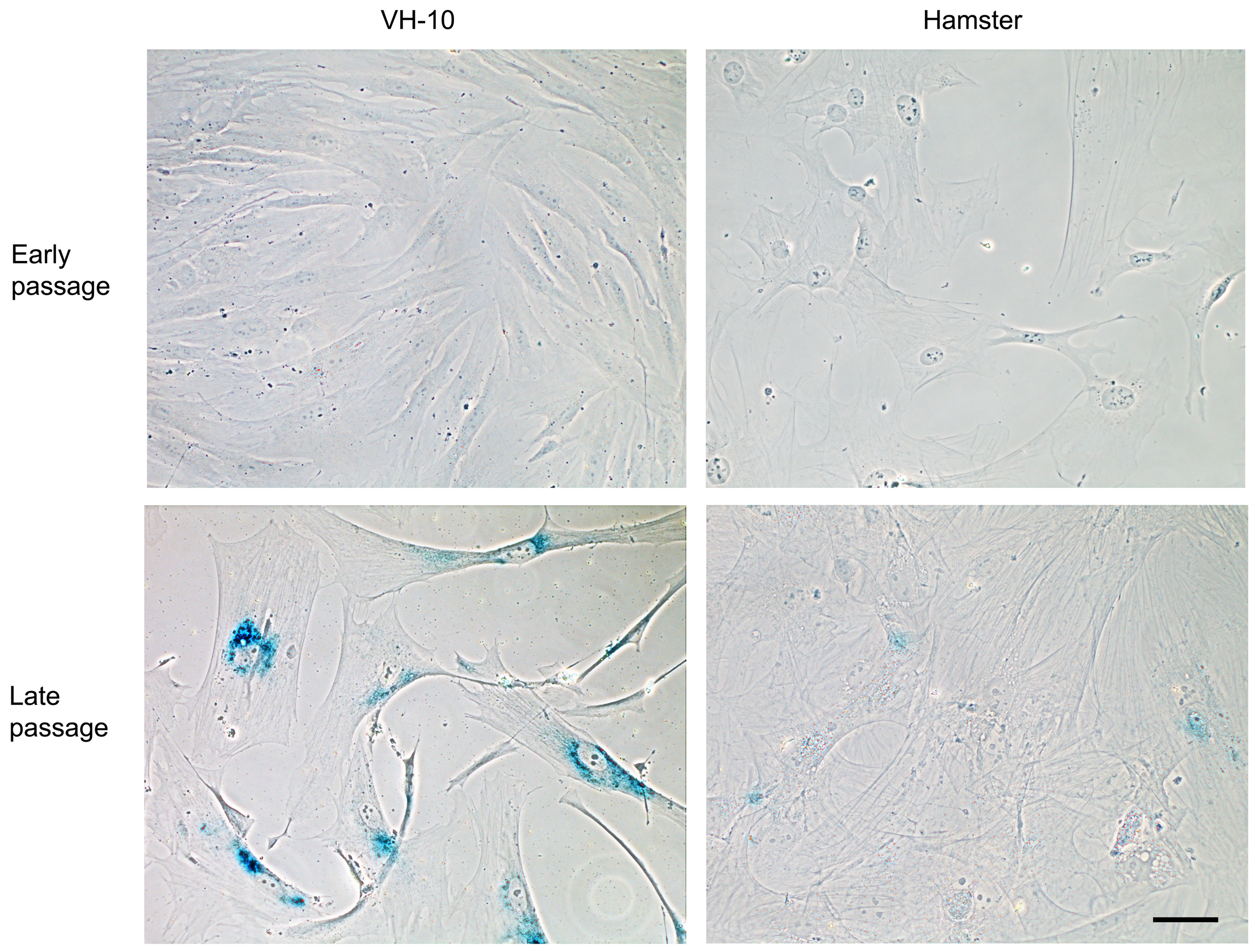

Supplement: Supplementary file 1 — 10.1186/s12867-015-0046-4 Detection of β-galactosidase activity in Syrian hamster early- and late-passage cells and human fibroblasts of different age. Phase contrast images of VH-10 human fibroblasts at the 19th and the 38th passages and Syrian hamster fibroblasts at the 1st and the 5th passages are shown. β-galactosidase is detected in blue color. Blue color is clearly expressed in senescent human cells, and only faint blue staining is observed in some of Syrian hamster presenescent cells. Bar is 50 µm. [file 12867_2015_46_MOESM1_ESM.tiff]

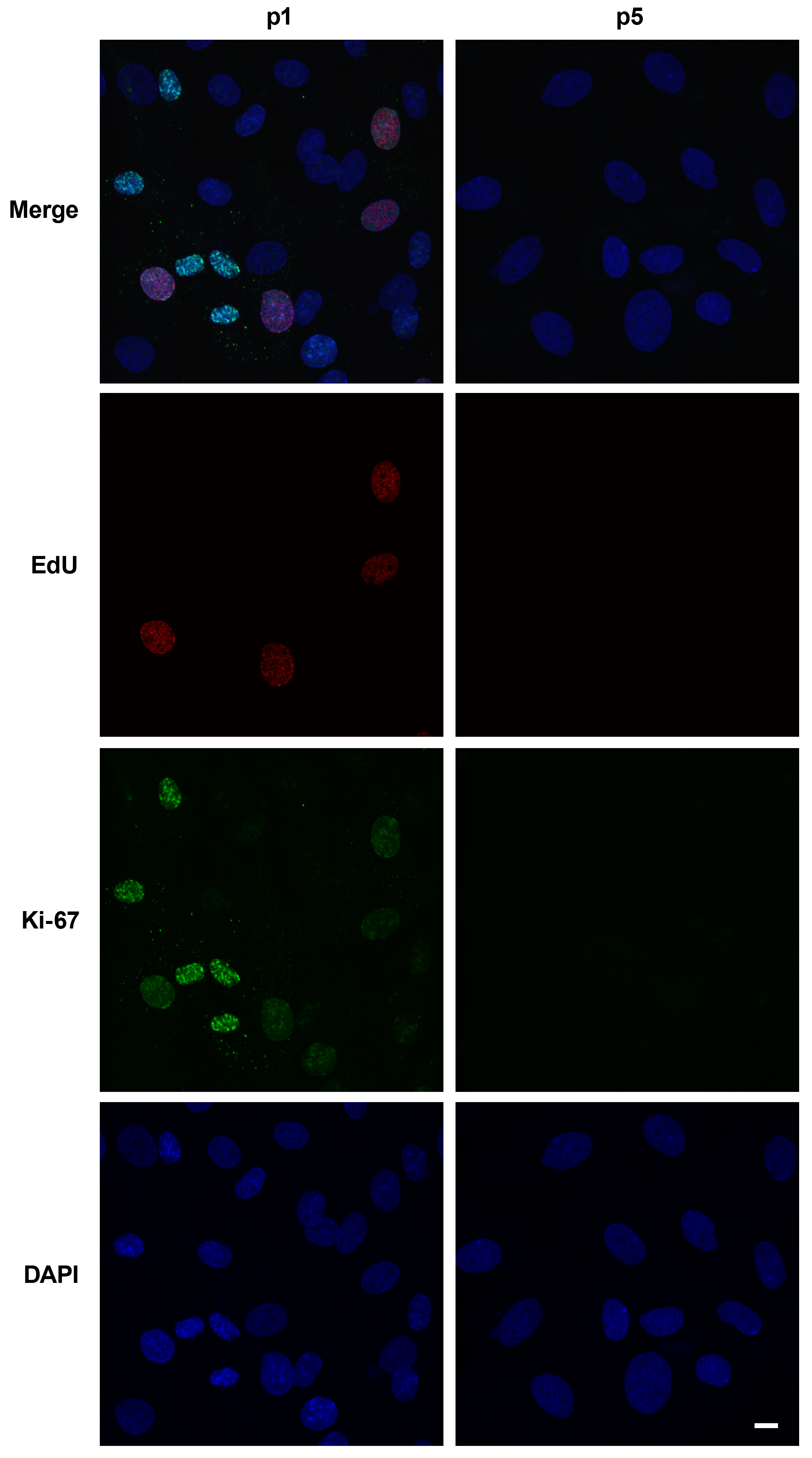

Supplement: Supplementary file 2 — 10.1186/s12867-015-0046-4 Proliferation is arrested in Syrian hamster fibroblasts at the 5th passage. EdU was incorporated for 30 min in untreated Syrian hamster cells at the 1st (p 1) and the 5th (p 5) passages. Proliferating cells at the 1st passage are Ki-67-positive, S-phase cells incorporate EdU, G0 cells are Ki-67- and EdU-negative. All cells at the 5th passage are Ki-67- and EdU-negative. DNA in cell nuclei is counterstained with DAPI. Bar is 10 µm. [file 12867_2015_46_MOESM2_ESM.tiff]

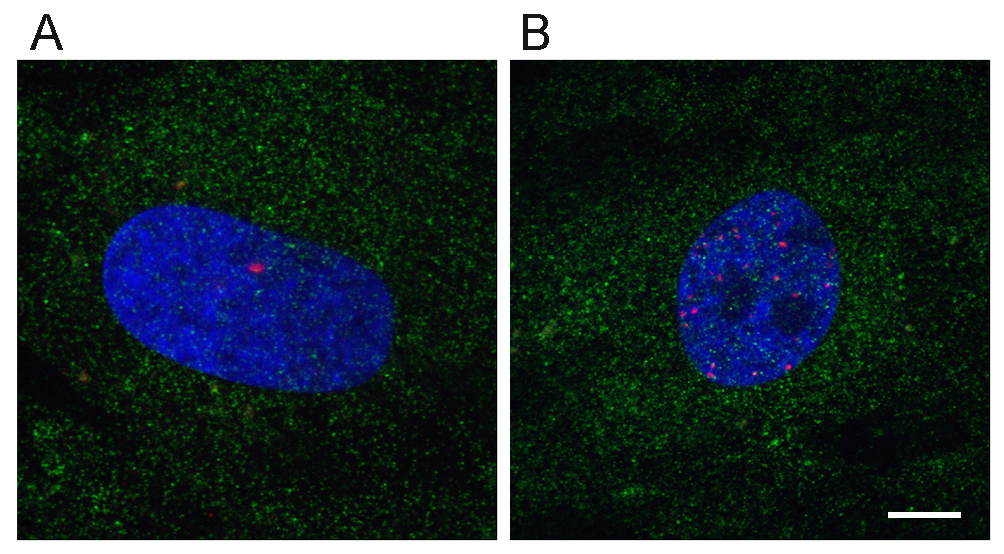

Supplement: Supplementary file 3 — 10.1186/s12867-015-0046-4 BL-hydrolase staining in Syrian hamster cells containing different numbers of DSBs. 1 h after BL treatment, cells containing different numbers of gH2AX foci demonstrate no visible difference in the density of cytoplasm staining by anti-BL-hydrolase antibody. Cells with one (A) and more than 20 (B) gH2AX foci are shown. Bar is 10 µm. [file 12867_2015_46_MOESM3_ESM.tiff]

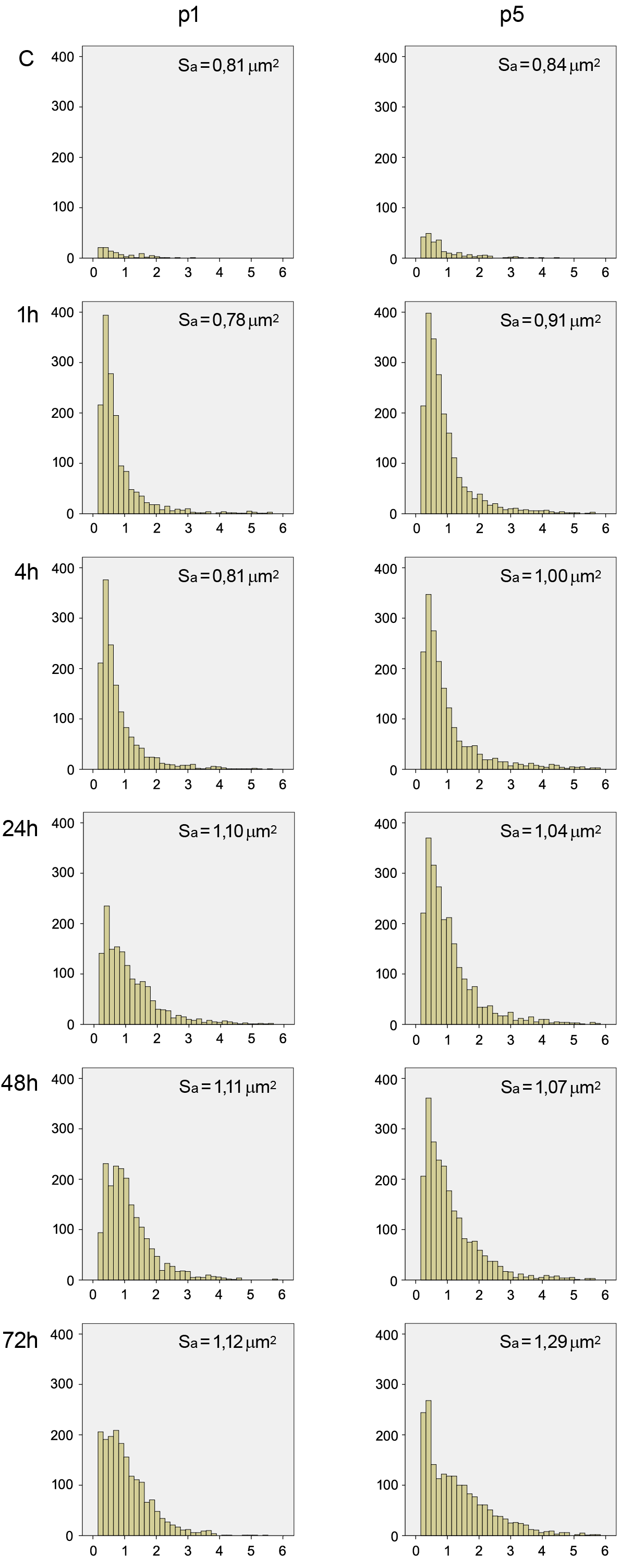

Supplement: Supplementary file 4 — 10.1186/s12867-015-0046-4 Histograms of projected gH2AX focus area distribution at different time points after BL treatment. Projected gH2AX focus area in young (p 1) and presenescent (p 5) Syrian hamster fibroblasts was measured after segmentation of images of maximal projections of confocal sections. Segmentation was performed using IPLab (Scananalytics) software with the same level of segmentation for all images. X axis—projected focus area (µm2), y axis—count of foci. Incubation time (1 h, 4 h, 24 h, 48 h, 72 h) after BL treatment is indicated, “C” indicates non-treated (control) cells. Sa—average projected area for indicated time point. 200 cells were taken for area measurements for each of histograms (with the exception for control histograms where 400 cells were taken). [file 12867_2015_46_MOESM4_ESM.tiff]
