# Supplementary material for: DNA double-strand break repair is impaired in presenescent Syrian hamster fibroblasts
Source: BMC Mol Biol. 2015 Oct 12;16:18. doi: 10.1186/s12867-015-0046-4 (PMC4601148; doi:10.1186/s12867-015-0046-4)
Supplement: Supplementary file 5 — 10.1186/s12867-015-0046-4 Colocalization of gH2AX with 53BP1, DNA-PK, pATM and p-ATM/ATR substrates in Syrian hamster fibroblasts at two time points after BL treatment. [file 12867_2015_46_MOESM5_ESM.pdf]

Table S1. Colocalization of gH2AX with 53BP1. DNA-PK, pATM and p-ATM/ATR substrates in Syrian hamster fibroblasts at two time points after BL treatment

Protein colocalized with gH2AX	Passage number	Time after BL (h)	Rr +/- StErr	p value	R +/- StErr	p value
53BP1	1	**0**	0.64 +/- 0.02	0.150	0.72 +/- 0.01	0.100
		**1**	0.68 +/- 0.02		0.75 +/- 0.02	
53BP1	5	**0**	0.46+/-0.03	<**0.001**	0.57+/- 0.02	<**0.001**
		**1**	0.72+/- 0.01		0.77 +/- 0.01	
pDNA-PK	1	**0**	0.51 +/- 0.01	0.144	0.60 +/- 0.01	0.448
		**1**	0.47 +/- 0.02		0.58 +/- 0.01	
pDNA-PK	5	**0**	0.35 +/- 0.02	<**0.001**	0.48 +/- 0.01	<**0.001**
		**1**	0.47 +/- 0.02		0.56 +/- 0.01	
pATM	1	**0**	0.65 +/- 0.01	0.491	0.72 +/-0.01	0.382
		**1**	0.64 +/- 0.01		0.71 +/-0.01	
pATM	5	**0**	0.59 +/- 0.02	0.058	0.67 +/- 0.01	0.247
		**1**	0.54 +/- 0.01		0.65 +/- 0.01	
pSub	1	**0**	0.48 +/- 0.02	**0.026**	0.54 +/- 0.02	0.070
		**1**	0.54+/- 0.02		0.60+/-0.01	
pSub	5	**0**	0.46+/- 0.03	0.540	0.50+/-0.03	0.698
		**1**	0.49+/- 0.02		0.56+/-0.02	

pSub – phospho-(Ser/Thr) ATM/ATR substrates.

Colocalization analysis was performed for G0 and G1 EdU-negative cells.

Rr - Pearson’s colocalization coefficient, R- Manders’ colocalization coefficient. In each variant, the coefficients are presented as mean +/- standard error for 25 cell nuclei. Student t test was used to compare values of coefficients obtained for the 1st and the 5th passages of Syrian hamster cells. Probability $p<0.05$ means that the difference between values is statistically significant.
